# Transforming Growth Factor-β1 in predicting early lung fibroproliferation in patients with acute respiratory distress syndrome

**DOI:** 10.1371/journal.pone.0206105

**Published:** 2018-11-05

**Authors:** Jean-Marie Forel, Christophe Guervilly, Catherine Farnarier, Stéphane-Yannis Donati, Sami Hraiech, Nicolas Persico, Jérôme Allardet-Servent, Benjamin Coiffard, Marc Gainnier, Anderson Loundou, Aude Sylvestre, Antoine Roch, Jeremy Bourenne, Laurent Papazian

**Affiliations:** 1 Médecine Intensive-Réanimation, Hôpital Nord, Assistance Publique–Hôpitaux de Marseille, Marseille, France; 2 CEReSS—Centre d'Etudes et de Recherches sur les Services de Santé et qualité de vie EA3279, Faculté de Médecine de Marseille Aix-Marseille Université, France; 3 Laboratoire d’Immunologie, Hôpital de la Conception, Assistance Publique–Hôpitaux de Marseille, Marseille, France; 4 Réanimation, Hôpital Sainte-Musse, Toulon, France; 5 Service d’Accueil des Urgences, Hôpital Nord, Assistance Publique–Hôpitaux de Marseille, Marseille, France; 6 Service de Réanimation, Hôpital Européen, Marseille, France; 7 Réanimation des Urgences et Médicale, Hôpital de la Timone, Assistance Publique–Hôpitaux de Marseille, Marseille, France; University of Western Ontario, CANADA

## Abstract

**Background:**

Fibroproliferative repair phase of the acute respiratory distress syndrome (ARDS) is followed by a *restitutio ad integrum* of lung parenchyma or by an irreversible lung fibrosis and patients’ death. Transforming Growth Factor-β1 (TGF-β1) is involved in collagen production and lung repair. We investigated whether alveolar TGF-β1 was associated with the presence of fibroproliferation and the outcome of ARDS patients.

**Methods:**

Sixty-two patients were included the first day of moderate-to-severe ARDS. Bronchoalveolar lavage fluid (BALF) was collected at day 3 (and day 7 when the patients were still receiving invasive mechanical ventilation) from the onset of ARDS. Survival was evaluated at day 60. TGF-β1 was measured by immunoassay. The patients were classified as having lung fibroproliferation when the alveolar N-terminal peptide for type III procollagen (NT-PCP-III) measured on day 3 was > 9 μg/L as recently reported. The main objective of this study was to compare the alveolar levels of total TGF-β1 according to the presence or not a lung fibroproliferation at day 3.

**Results:**

Forty-three patients (30.6%) presented a fibroproliferation at day 3. BALF levels of total TGF-β1 were not statistically different at day 3 (and at day 7) according to the presence or not lung fibroproliferation. Mortality at day 60 was higher in the group of patients with fibroproliferation as compared with patients with no fibroproliferation (68.4% vs. 18.6% respectively; *p* < 0.001). Total TGF-β1 measured on BALF at day 3 was not associated with the outcome. Multiple logistic regression showed that the presence of lung fibroproliferation was associated with death. In contrast, TGF-β1 was not independently associated with death.

**Conclusions:**

Pulmonary levels of TGF-β1 during the first week of ARDS were not associated nor with the presence of fibroproliferation neither with death. TGF-β1 should not be used as a biomarker to direct anti-fibrotic therapies.

## Introduction

Acute respiratory distress syndrome (ARDS) is associated with a mortality rate of 30−60%.[1, 2] Results from histopathological studies classically show two successive periods during ARDS: an early inflammatory phase followed by a fibroproliferative repair phase, with cell proliferation and deposition of matrix proteins leading to the resolution of ARDS or to irreversible lung fibrosis and death.[3, 4, 2]

Transforming Growth Factor-β1 (TGF-β1) plays a pivotal role in lung repair and fibroproliferative processes characterized by the collagen synthesis.[5, 6] TGF-β1 is secreted as a latent complex and must be released from its propeptide to acquire its biological activity (active TGF-β1).[7] Active TGF-β1 is a potent direct stimulator of collagen production.[8–10] TGF-β1 overexpression results in fibroblast migration and proliferation with increased deposition of extracellular matrix.[11, 12] Budinger *et al*. showed that bronchoalveolar lavage fluid (BALF) from ARDS patients, as well as exogenous TGF-β1, activate the human procollagen I promoter.[13] Experimental studies suggest that TGF-β1 is involved early in the course of acute lung injury.[14–19] The inflammatory properties of TGF-β1 could participate in processes observed early in the course of ARDS such as lung cells recruitment, alveolar flooding and cytokine release.[15, 17, 20, 21] However, little is known about the early changes in TGF-β1 levels and their prognostic values in human ARDS.

Procollagen Type III-N-terminal peptide (NT-PCP-III), a peptide released during the conversion of type III procollagen to type III collagen, is a marker of fibroblast activity, collagen synthesis and lung fibrosis.[22, 23] NT-PCP-III is increased in alveolar fluid and serum during lung fibrosis and fibroproliferative-established ARDS.[24–27] Pulmonary levels of NT-PCP-III are also elevated from the onset of ARDS, suggesting that fibroproliferation is involved early in the lung repair process and occurs simultaneously with the inflammatory phase of ARDS.[28–31] Early fibroproliferation in ARDS patients with higher pulmonary levels of NT-PCP-III has been shown to be independently associated with death.[27, 31, 32, 33] It has also been recently reported that NT-PCP-III is a valid biomarker of lung fibroproliferation [34].

Because TGF-β1 could play a central role in the pathophysiology of ARDS during the early phase, the objectives of the present study were to evaluate if alveolar TGF-β1 obtained from BALF analysis was associated with fibroproliferation (assessed by lung production of NT-PCP-III) and with the outcome. The identification of reliable biomarkers is of paramount importance to guide anti-inflammatory treatments and/or therapeutics that modulate fibroproliferation such as corticosteroids.

## Materials and methods

The study was approved by the local ethics committee (Comité Consultatif de Protection des Personnes dans la Recherche Biomédicale (CCPPRB) de Marseille 1) and was registered (ClinicalTrials.gov NCT00440882). Written informed consent was obtained from patients and/or next of kin prior to enrollment.

### Patients

Patients over 18 years of age were included within the first 24 h of the onset of moderate-to-severe ARDS if they presented a partial pressure of arterial oxygen/fraction of inspired oxygen ratio (PaO_2_/FIO_2_) <200 mmHg at a positive end-expiratory pressure (PEEP) ≥ 5 cm H_2_O [35]. Some of these patients (n = 9) have also been included in the validation cohort of NT-PCP-III previously reported [34]. The exclusion criteria were as follows: pregnancy, chronic interstitial or fibrotic lung diseases, liver cirrhosis, neutropenia ≤ 1 G/L, corticosteroid (>200 mg/day of hydrocortisone or equivalent beginning at least 2 weeks before inclusion), immunosuppressive therapy within the last 30 days, presence of an advanced directive to withhold life-sustaining treatment, persistent (more than 4 h) PaO_2_/FIO_2_ <70 mmHg despite maximal treatment (safety criteria for BAL). Written informed consent was obtained from patients and/or next of kin prior to enrollment.

### Study protocol

ARDS patients were ventilated according to a lung-protective strategy (4 ≤ tidal volume ≤ 8 mL/Kg predicted body weight and plateau pressure ≤ 30 cm H_2_O). PEEP and FIO_2_ were set according to the algorithm of the National Institutes of Health ARDS Network.[1] Patients with septic shock were treated with 200 mg/day of hydrocortisone for at least the first 5 days. Clinical data, respiratory parameters, and sepsis-related organ failure assessment score (SOFA)[36] were evaluated on days 1 (inclusion), 3 and 7. Survival was evaluated on day 60. Ventilator-free days and alive were evaluated both at day 28 and day 60.

### Blood and bronchoalveolar lavage fluid collection

BALF samples were collected on day 3 and 7 from ARDS onset if the patients were still receiving invasive mechanical ventilation. BALF was always obtained from the same territory, corresponding to the most infiltrated lung area on chest X-rays. Sterile saline was instilled in an aliquot of 50 mL. A second aliquot of 50 mL was used if the recovered lavage fluid was lower than 10 mL. After centrifugation, BALF aliquots were stored at -80°C until their analysis.

### Biological analyses

TGF-β1 was measured using a specific ELISA kit (Quantikine, R&D Systems, MN, USA). This immunoassay was performed directly on the BALF samples to measure the spontaneous free TGF-β1 (free TGF-β1). The latent form of TGF-β1 was measured following an acid activation and neutralization protocols in agreement with the manufacturer's instructions. This acidification induced an activation of the latent form of TGF-β1 and allowed the measure of the both latent and spontaneous free forms (total TGF-β1) in BALF. Assay sensitivity limits was less than 2 pg/mL in BALF. BALF levels of NT-PCP-III were measured by radioimmunoassay (Orion Diagnostica, Finland). This kit is based on a competitive technique and detects intact NT-PCP-III. The assays were performed in agreement with the manufacturer's instructions. The detection limit was 0.3 μg/L.

### Definition of lung fibroproliferation

The patients were classified as having lung fibroproliferation when the NT-PCP-III measured on day 3 was > 9 μg/L on BALF as recently reported [34].

### Objectives of the study

The main objective of this study was to compare the alveolar levels of total TGF-β1 according to the presence or not a lung fibroproliferation at day 3. Secondary objectives included the diagnostic value of BALF levels of free TGF-β1 in predicting lung fibroproliferation at day 3, and the association between BALF levels of total TGF-β1 and the outcome.

### Statistics

Descriptive variables of the population were compared by the Fisher’s exact test for categorical variables and the Student’s t test for normally distributed continuous variables. We used the Mann-Whitney test for the non-normally distributed variables (Kolmogorov-Smirnov approach) as total and free TGF-β1 levels. Two-tailed tests were used. Non-normally distributed variables were reported as median values [interquartile range] and normally distributed variables are reported as mean (SD). A multiple logistic regression analysis was done in order to identify the factors related to the outcome. A *p* value of ≤ 0.05 was considered to be statistically significant. Statistics were performed using SPSS 20.0 software (SPSS Inc., IL, USA).

## Results

### Patients

**Fig 1:**
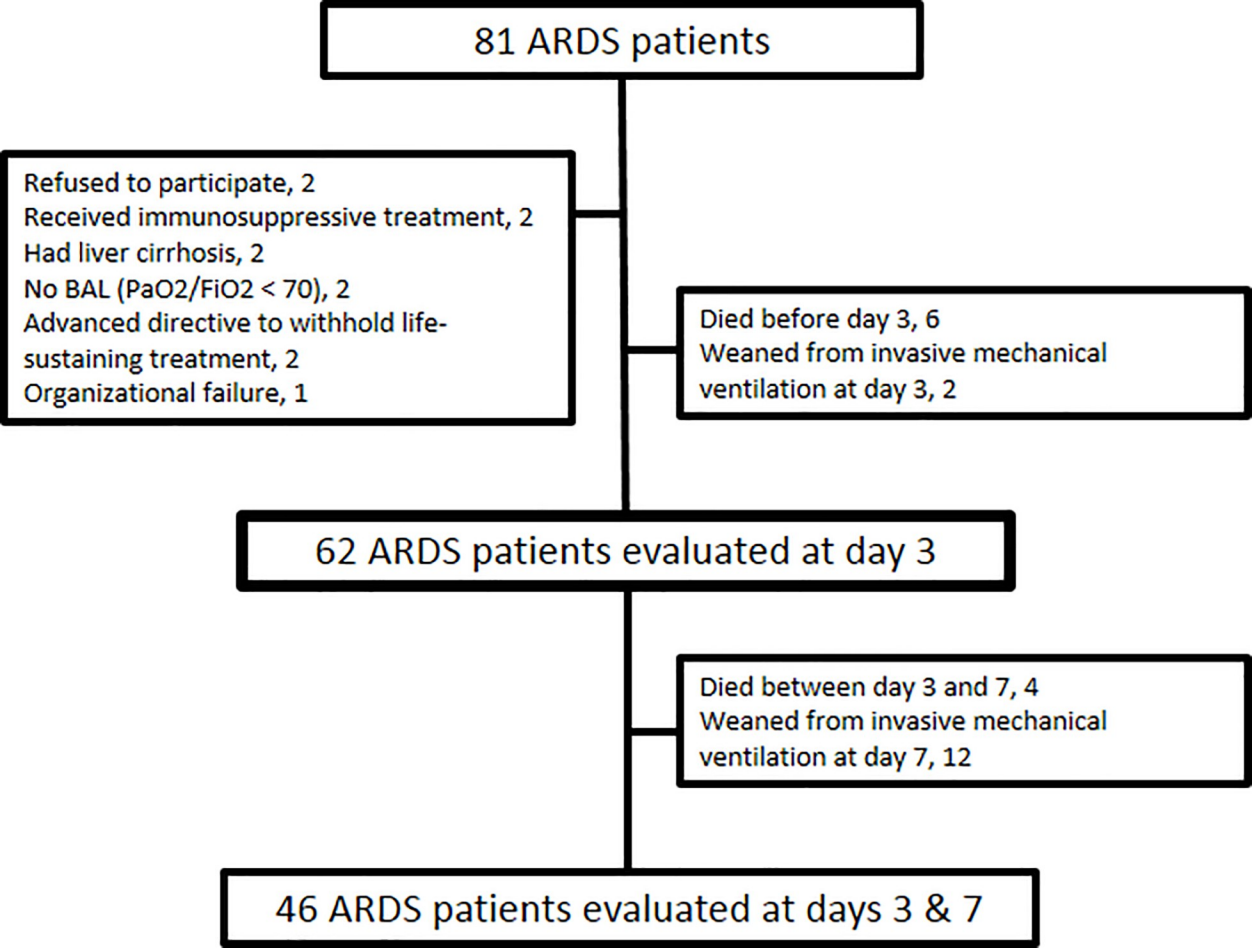
Flow chart of the study. Day 1 is the day of inclusion; ARDS: acute respiratory distress syndrome; PaO2/FIO2, partial pressure of arterial oxygen/fraction of inspired oxygen ratio; BALF: bronchoalveolar lavage.

Eighty-one consecutive ARDS patients were screened in three intensive care units (34 beds) (Fig 1). The BALF samples were obtained from all 62 prospectively included patients. The baseline characteristics of these 62 patients are shown in Tables 1 and 2. Direct lung injury (such as pneumonia and aspiration pneumonitis) was the main mechanism of ARDS (85.5% of the patients). Forty-three patients (30.6%) presented a fibroproliferation on day 3. In Table 2 are presented the respiratory parameters and ventilator settings on days 1, 3 and 7 of ARDS according to the presence/absence of fibroproliferation. Interestingly, driving pressure and plateau pressure were higher in the fibroproliferation group on days 3 and 7, but not on day 1. Overall mortality at day 60 was 33.9% (n = 21). Mortality at day 60 was higher in the group of patients from the fibroproliferation group (68.4% vs. 18.6% in the no fibroproliferation group; *p* < 0.001).

**Table 1:** Characteristics on inclusion and outcome of the patients.

	All (n = 62)	No fibroproliferation (n = 43)	Fibroproliferation (n = 19)	*p* value
**Age, years**	59 ± 15	56 ± 14	65 ± 14	0.025
**Men, n (%)**	47 (75.8)	26 (78.8)	12 (92.3)	0.276
**SAPS II score**	47.4 ± 14.7	47.8 ± 15.9	46.5 ± 11.9	0.754
**SOFA score**	9.5 ± 3.2	9.6 ± 3.5	9.3 ± 2.4	0.698
**Cause of ARDS, n (%)**				0.117
**Pneumonia**	37 (59.7)	21 (48.8)	16 (84.2)	
**Aspiration**	14 (22.6)	13 (30.2)	1 (5.3)	
**Extra-pulmonary infection**	5 (8.1)	4 (9.3)	1 (5.3)	
**Pancreatitis**	1 (1.6)	1 (2.3)	0 (0)	
**Miscellaneous**	5 (8.1)	4 (9.3)	1 (5.3)	
**ARDS with direct lung injury, n (%)**	53 (85.5)	36 (87.7)	17 (89.5)	0.553
**Lung Injury Severity Score**	2.86 ± 0.48	2.85 ± 0.49	2.88 ± 0.48	0.834
**Hydrocortisone, n (%)**	48 (77.4)	30 (69.8)	18 (94.7)	0.046
**Ventilator free day 28, days**	1 [0–12]	4 [0–18]	0 [0–0]	0.006
**Ventilator free day 60, days**	32 [0–44]	36 [0–50]	0 [0–0]	0.002
**ICU-free day 60, days**	26 [0–39]	32 [0–43]	0 [0–0]	0.001
**Death at day 60, n (%)**	21 (33.9)	8 (18.6)	13 (68.4)	< 0.001

Values are expressed as means ± SD, number of cases (%) or median [IQR]

ARDS, acute respiratory distress syndrome; SAPS II, simplified acute physiology score; SOFA, sepsis-related organ failure assessment score; Hydrocortisone, septic shock patients treated by hydrocortisone ≥ 200 mG/kG/day during at least 5 days after inclusion

**Table 2:** Evolution of respiratory parameters and ventilator settings according to the presence/absence of lung fibroproliferation (alveolar N-terminal peptide for type III procollagen > 9 μg/L).

	ARDS Day 1		ARDS Day 3		ARDS Day 7	
	No fibroproliferation (N = 43)	Fibroproliferation (N = 19)	No fibroproliferation (n = 43)	Fibroproliferation (n = 19)	No fibroproliferation (N = 33)	Fibroproliferation (N = 13)
**Tidal volume, mL/kg PBW**	6.6 ± 0.9	6.6 ± 0.8	6.8 ± 1.0	6.9 ± 1.3	7.4 ± 1.8	6.9 ± 2.3
**Respiratory rate, c/min**	21.3 ± 5.7	25.5 ± 6.5 *	22.3 ± 6.2	26.4 ± 5.7 #	23.8 ± 8.1	30.8 ± 8.1 †
**Total PEEP, cmH**_**2**_**O**	12.6 ± 3.1	12.1 ± 2.6	11.8 ± 3.5	11.7 ± 2.4	9.9 ± 3.9	11.5 ± 3.7
**Plateau pressure, cmH**_**2**_**O**	24.9 ± 5.0	26.7 ± 4.3	24.4 ± 5.3	27.7 ± 5.1 #	22.7 ± 7.0	28.9 ± 6.6 †
**Driving pressure, cmH**_**2**_**O**	12.3 ± 3.9	14.6 ± 4.9	12.4 ± 4.1	16.1 ± 5.2 #	12.5 ± 5.5	17.1 ± 5.6 †
**Tidal compliance rs, mL/ cmH**_**2**_**O**	39.2 ± 18.5	32.8 ± 11.3	38.9 ± 14.4	31.4 ± 12.9	45.8 ± 27.8	33.6 ± 27.7
**FiO**_**2**_	0.68 ± 0.10	0.76 ± 0.19	0.55 ± 0.15	0.61 ± 0.16	0.48 ± 0.14	0.68 ± 0.22 †
**PaO**_**2**_**/FiO**_**2**_**, mmHg**	122.9 ± 33.3	112.1 ± 29.0	170.6 ± 67.9	135.9 ± 38.6 #	200.8 ± 77.3	127.6 ± 57.6 †
**PaCO**_**2**_**, mmHg**	46.9 ± 13.3	53 ± 14	42.6 ± 8.1	48.2 ± 9.9 #	43.1 ± 10.1	52.7 ± 15.4 †
**pH**	7.33 ± 0.10	7.28 ± 0.10	7.39 ± 0.07	7.34 ± 0.09 #	7.43 ± 0.08	7.37 ± 0.11

Values are expressed as means ± SD.

*: p < 0.05 for comparison on day 1

**#**: p < 0.05 for comparison of ventilator parameters on day 3

**†**: p < 0.05 for comparison of ventilator parameters on day 7

ARDS, acute respiratory distress syndrome; PaO2/FIO2: partial pressure of arterial oxygen/fraction of inspired oxygen ratio; PBW: predicted body weight; rs: respiratory system; plateau pressure was measured during a 1 second end-inspiratory pause; Total PEEP: total positive end-expiratory pressure was measured during a 5 seconds end-expiratory pause.

### Transforming growth factor-β1 levels in predicting early lung fibroproliferation

**Fig 2:**
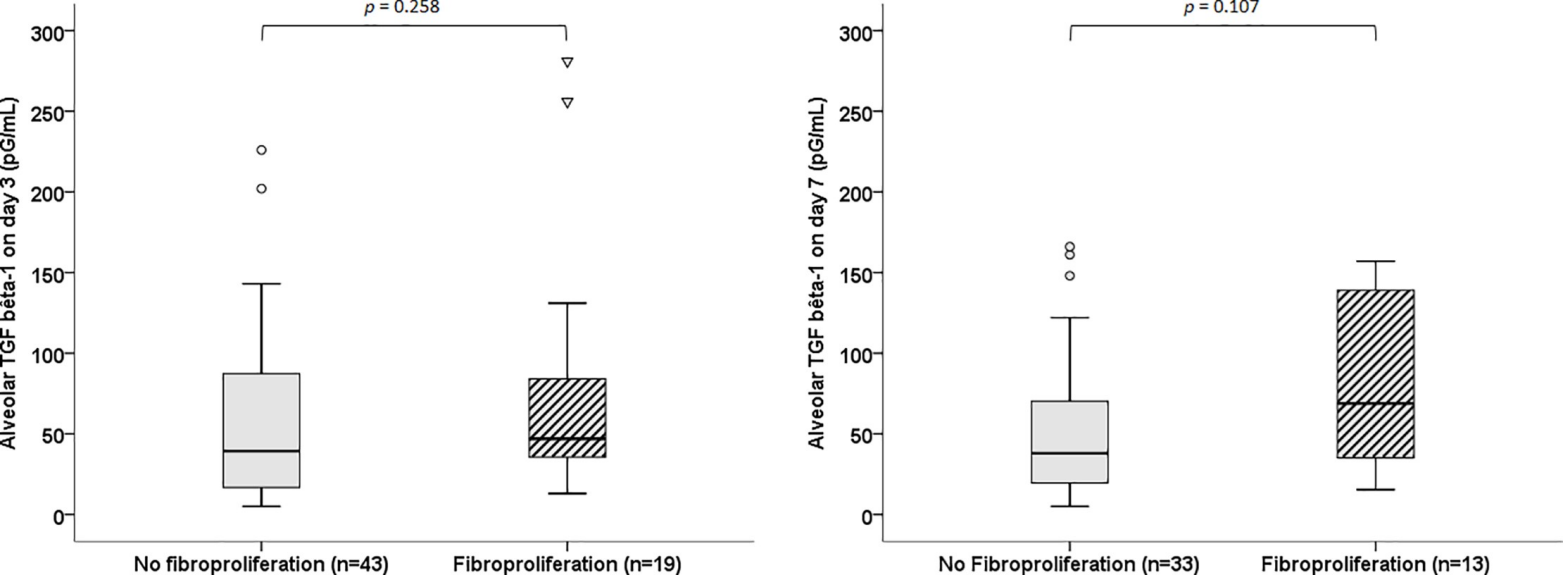
Bronchoalveolar lavage fluid levels of total TGF-β1 on day 3 (left panel) and day 7 (right panel) in ARDS patients according to lung fibroproliferation evaluated on day 3.

BALF levels of total TGF-β1 were not statistically different between patients presenting or not lung fibroproliferation both at day 3 and at day 7 (Fig 2). The ROC curves (S1 Fig) showed that the diagnostic performance of BALF levels of TGF-β1 for the identification of lung fibroproliferation was poor. Furthermore, there was no difference regarding BALF free TGF-β1 levels in patients with and without lung fibroproliferation both at day 3 and day 7 (S1 Table).

In the specific subgroup analysis of the 9 patients which have been included in the validation cohort of PCIII previously reported [34] with an available open lung biopsy, 5 patients with histologically documented lung fibrosis were reported. No significant difference was observed between BALF total TGF-β1 levels measured on days 3 and 7 (S2 Fig).

### Transforming growth factor-β1 levels in ARDS according to the outcome

Total TGF-β1 measured on BALF on day 3 was not associated with the outcome (Table 3 and S3 Fig). In contrast, the presence of lung fibroproliferation as assessed by NT-PCP-III determined on BALF obtained at day 3 was strongly and independently associated with day-60 mortality (Table 3). The SOFA score evaluated on day 3 was also independently associated with day-60 mortality while other factors such as driving pressure or age were not (Table 3).

**Table 3:** Factors evaluated on ARDS day 3 and associated with mortality at day 60.

	Survivors (N = 41)	Non-survivors (N = 21)	Univariate	HR (95% CI)	Multivariate
**Age, years**	55.9 ± 14.8	65.6 ± 11.9	0.012	1.022 (0.929–1.124)	0.661
**Men, n (%)**	31 (75.6)	16 (76.2)	0.960	-	-
**SOFA score**	7.3 ± 3.5	11.0 ± 3.3	<0.001	1.463 (1.064–2.012)	0.019
**LIS Score**	2.43 ± 0.56	2.72 ± 0.68	0.086	0.442 (0.043–4.506)	0.491
**Tidal volume, mL/kg PBW**	6.8 ± 1.0	6.9 ± 1.2	0.597	-	-
**Respiratory rate, c/min**	21.5 ± 5.7	28.0 ± 5.4	<0.001	-	-
**Total PEEP, cmH**_**2**_**O**	11.7 ± 3.4	12.1 ± 2.7	0.619	-	-
**Plateau pressure, cmH**_**2**_**O**	24.4 ± 5.2	28.3 ± 5.2	0.018	-	-
**Driving pressure, cmH**_**2**_**O**	12.7 ± 4.3	16.3 ± 5.1	0.013	1.163 (0.927–1.457)	0.192
**Tidal compliance rs, mL/ cmH**_**2**_**O**	38.9 ± 13.9	29.9 ± 13.4	0.041	-	-
**PaO**_**2**_**/FiO**_**2**_**, mmHg**	171.6 ± 65.9	131.7 ± 41.2	0.021	-	-
**PaCO**_**2**_**, mmHg**	43.9 ± 8.7	45.5 ± 9.8	0.519	-	-
**pH**	7.39 ± 0.06	7.32 ± 0.09	0.001	0.001 (0.0001–350.827)	0.170
**Lung Fibroproliferation, n (%)**	6 (14.6)	13 (61.9)	<0.001	24.236 (1.684–349.482)	0.019
**Total TGF-β1, pg/L**	60.6 ± 52.6	76.6 ± 75.5	0.332	1.003 (0.986–1.019)	0.768

Values are expressed as means ± SD or number of cases (%). ARDS, acute respiratory distress syndrome; SOFA, sepsis-related organ failure assessment score; PaO_2_/FIO_2_, partial pressure of arterial oxygen/fraction of inspired oxygen ratio; PBW, predicted body weight; plateau pressure measured during a 1-second end-inspiratory pause; Total PEEP, total positive end-expiratory pressure was measured during a 5-second end-expiratory pause. Tidal compliance rs, Plateau Pressure and Driving Pressure were collinear. Driving Pressure was introduced in the multiple logistic regression. Respiratory Rate and pH were collinear. pH was introduced in the model. SOFA score and PaO_2_/FiO_2_ were collinear. SOFA score was introduced in the model

## Discussion

In this prospective clinical study, we observed that alveolar fluid levels of total TGF-β1 were not associated with the presence of lung fibroproliferation early in the course of ARDS patients. Pulmonary levels of TGF-β1 were not different between ARDS survivors and nonsurvivors. In contrast, the presence of lung fibroproliferation as assessed by an elevated level of NT-PCP-III determined on BALF early in the course of ARDS was strongly associated with mortality.

We did not observe any difference in alveolar levels of free and total TGF-β1 between survivors and nonsurvivors in this group of 62 ARDS patients. In a previous study about the prognostic value of free TGF-β1 levels in BALF performed in 29 ARDS patients, Budinger *et al*. found a trend to higher BALF levels of TGF-β1 in ARDS nonsurvivors as compared to survivors but the difference did not reach the significance (*p* = 0.14).[13] TGF-β1 mediates far-ranging biological processes including cell growth, inflammation, angiogenesis, tumorogenesis, morphogenesis, fibrogenesis and tissue repair.[5, 37, 38] The exact biological role of TGF-β1 in lung pathophysiology has not been clearly identified. A beneficial role is observed in experimental studies where TGF-β1 is expressed at high levels during normal lung development.[5, 39] Moreover, TGF-β1 is also involved in normal tissue repair following lung injury.[40, 41] Nevertheless, a harmful role of TGF-β1 in the development of acute lung injury has been reported.[17, 15] These pleiotropic functions of TGF-β1 and its complex regulation could limit its prognostic value. The relation between TGF-β1, collagen synthesis and ARDS outcome remains complex and the weak relationships between alveolar levels of TGF-β1 and NT-PCP-III, observed in the current study, seem to suggest that the NT-PCP-III synthesis depends of other mediators than TGF-β1.

### Study limitations

In our study, the main underlying conditions responsible for ARDS were pneumonia (59.7%) and gastric aspiration pneumonitis (22.6%). This could have influenced the TGF-β1 levels observed in our population because Buhling *et al*. showed that bacterial pneumonia increased TGF-β1 levels.[42] Conclusions could have been different in patients with ARDS from extrapulmonary causes.

TGF-β exists in three isoforms (β1, 2 and 3)[5, 37] but in the present study, TGF-β2 and 3 were not measured. There is evidence that TGF-β2 and β3 exert significant *in vitro* pro-fibrotic activity and could also drive fibrogenesis in the lung.[43] Further studies are needed to determine if TGF-β2 and β3 could be associated with fibroproliferation and mortality in ARDS patients. Moreover, the detection of TGF-β1 used an immunoassay measuring the level of free TGF-β1 and, after in vitro activation of latent (complexed) forms of TGF-β1, the total TGF-β1. This assay quantified the amount of TGF-β1 but did not measure the biological activity of the free TGF-β1. The interpretation of the present results must consider this limitation.

Fibroproliferation was based on a NT-PCP-III threshold at 9 μg/L. This threshold has been evaluated in a recently published study where 32 ARDS patients were included [34]. A threshold at 9 μg/L was compared with histological analysis of lung parenchyma. The diagnostic accuracy of a NT-PCP-III higher than 9 μg/L to predict the presence of lung fibroproliferation was 90.6 (95% CI, 75.8–96.8)%. In the present study, we showed that our results were reliable by comparing biopsy results with BALF NT-PCP-III and TGF-β determinations in a subset of 9 patients. Moreover, we reported here that TGF-β was not associated with the outcome, whereas the presence of lung fibroproliferation was linked to mortality.

## Conclusions

We showed that alveolar fluid levels of both total and free TGF-β1 evaluated early in the course of ARDS were not associated with the presence of lung fibroproliferation. Moreover, the alveolar fluid levels of total and free TGF-β1 were not associated with an increased risk of death in ARDS patients. We confirmed that the presence of fibroproliferation assessed by elevated pulmonary levels of NT-PCP-III measured within the first 7 days of the onset of ARDS was independently associated with death.

## Ethics approval and consent to participate

The study was approved by the local ethics committee (Comité Consultatif de Protection des Personnes dans la Recherche Biomédicale (CCPPRB) de Marseille 1) and was registered (ClinicalTrials.gov NCT00440882).

## Availability of data and materials

The datasets used and/or analysed during the current study are available from the corresponding author on reasonable request.

## Supporting information

S1 Fig ROC curves for BALF levels of TGF-β1 for the identification of lung fibroproliferation. (PDF)

S2 Fig Bronchoalveolar lavage fluid levels of TGF-β1 in ARDS patients according to lung fibroproliferation evaluated by the level of alveolar NT-PCP-III (left panel) and histology (right panel).
(PDF)

S3 Fig Bronchoalveolar lavage fluid levels of Transforming Growth Factor-β1 levels in ARDS according to the outcome. (PDF)

S1 Table Alveolar Free TGF β-1 according to the presence or not of lung fibroproliferation. (DOCX)

S1 Database (XLSX)

## Abbreviations

ARDS: Acute respiratory distress syndrome
BAL: bronchoalveolar lavage
BALF: bronchoalveolar lavage fluid
FIO_2_: fraction of inspired oxygen
ICU: Intensive Care Unit
LIS: lung injury severity score
NT-PCP-III: Procollagen Type III-N-terminal peptide
PaO_2_/FIO_2_: partial pressure of arterial oxygen/fraction of inspired oxygen ratio
PBW: predicted body weight
PEEP: positive end-expiratory pressure
SOFA: sepsis-related organ failure assessment score
TGF-β1: Transforming Growth Factor-β1
VFD: Ventilator-free days

## References

[1] Ventilation with lower tidal volumes as compared with traditional tidal volumes for acute lung injury and the acute respiratory distress syndrome. The Acute Respiratory Distress Syndrome Network. N Engl J Med. 2000;342(18):1301–8. 10.1056/NEJM200005043421801 10793162

[2] Ware LB, Matthay MA. The acute respiratory distress syndrome. N Engl J Med. 2000;342(18):1334–49. 10.1056/NEJM200005043421806 10793167

[3] Anderson WR, Thielen K. Correlative study of adult respiratory distress syndrome by light, scanning, and transmission electron microscopy. Ultrastruct Pathol. 1992;16(6):615–28. 1448881 10.3109/01913129209023751

[4] Martin C, Papazian L, Payan MJ, Saux P, Gouin F. Pulmonary fibrosis correlates with outcome in adult respiratory distress syndrome. A study in mechanically ventilated patients. Chest. 1995;107(1):196–200. 7813276 10.1378/chest.107.1.196

[5] Bartram U, Speer CP. The role of transforming growth factor beta in lung development and disease. Chest. 2004;125(2):754–65. 14769761 10.1378/chest.125.2.754

[6] Gauldie J, Bonniaud P, Sime P, Ask K, Kolb M. TGF-beta, Smad3 and the process of progressive fibrosis. Biochem Soc Trans. 2007;35(Pt 4):661–4. 10.1042/BST0350661 17635115

[7] Gleizes PE, Munger JS, Nunes I, Harpel JG, Mazzieri R, Noguera I et al TGF-beta latency: biological significance and mechanisms of activation. Stem Cells. 1997;15(3):190–7. 10.1002/stem.150190 9170210

[8] Raghu G, Masta S, Meyers D, Narayanan AS. Collagen synthesis by normal and fibrotic human lung fibroblasts and the effect of transforming growth factor-beta. Am Rev Respir Dis. 1989;140(1):95–100. 10.1164/ajrccm/140.1.95 2751176

[9] Coker RK, Laurent GJ, Shahzeidi S, Lympany PA, du Bois RM, Jeffery PK et al Transforming growth factors-beta 1, -beta 2, and -beta 3 stimulate fibroblast procollagen production in vitro but are differentially expressed during bleomycin-induced lung fibrosis. Am J Pathol. 1997;150(3):981–91. 9060836 PMC1857875

[10] Leask A, Abraham DJ. TGF-beta signaling and the fibrotic response. Faseb J. 2004;18(7):816–27. 10.1096/fj.03-1273rev 15117886

[11] Westergren-Thorsson G, Hernnas J, Sarnstrand B, Oldberg A, Heinegard D, Malmstrom A. Altered expression of small proteoglycans, collagen, and transforming growth factor-beta 1 in developing bleomycin-induced pulmonary fibrosis in rats. J Clin Invest. 1993;92(2):632–7. 10.1172/JCI116631 7688761 PMC294895

[12] Gauldie J, Jordana M, Cox G. Cytokines and pulmonary fibrosis. Thorax. 1993;48(9):931–5. 8236078 10.1136/thx.48.9.931 PMC464781

[13] Budinger GR, Chandel NS, Donnelly HK, Eisenbart J, Oberoi M, Jain M. Active transforming growth factor-beta1 activates the procollagen I promoter in patients with acute lung injury. Intensive Care Med. 2005;31(1):121–8. 10.1007/s00134-004-2503-2 15565360 PMC7095267

[14] Shenkar R, Coulson WF, Abraham E. Anti-transforming growth factor-beta monoclonal antibodies prevent lung injury in hemorrhaged mice. Am J Respir Cell Mol Biol. 1994;11(3):351–7. 10.1165/ajrcmb.11.3.8086171 8086171

[15] Pittet JF, Griffiths MJ, Geiser T, Kaminski N, Dalton SL, Huang X et al TGF-beta is a critical mediator of acute lung injury. J Clin Invest. 2001;107(12):1537–44. 10.1172/JCI11963 11413161 PMC200192

[16] Fahy RJ, Lichtenberger F, McKeegan CB, Nuovo GJ, Marsh CB, Wewers MD. The acute respiratory distress syndrome: a role for transforming growth factor-beta 1. Am J Respir Cell Mol Biol. 2003;28(4):499–503. 10.1165/rcmb.2002-0092OC 12654639

[17] Dhainaut JF, Charpentier J, Chiche JD. Transforming growth factor-beta: a mediator of cell regulation in acute respiratory distress syndrome. Crit Care Med. 2003;31(4 Suppl):S258–64. 12682450 10.1097/01.CCM.0000057901.92381.75

[18] Wesselkamper SC, Case LM, Henning LN, Borchers MT, Tichelaar JW, Mason JM et al Gene expression changes during the development of acute lung injury: role of transforming growth factor beta. Am J Respir Crit Care Med. 2005;172(11):1399–411. 10.1164/rccm.200502-286OC 16100012 PMC2718437

[19] Jenkins RG, Su X, Su G, Scotton CJ, Camerer E, Laurent GJ et al Ligation of protease-activated receptor 1 enhances alpha(v)beta6 integrin-dependent TGF-beta activation and promotes acute lung injury. J Clin Invest. 2006;116(6):1606–14. 10.1172/JCI27183 16710477 PMC1462943

[20] Hurst VI, Goldberg PL, Minnear FL, Heimark RL, Vincent PA. Rearrangement of adherens junctions by transforming growth factor-beta1: role of contraction. Am J Physiol. 1999;276(4 Pt 1):L582–95. 10198356 10.1152/ajplung.1999.276.4.L582

[21] Wahl SM. Transforming growth factor-beta: innately bipolar. Curr Opin Immunol. 2007;19(1):55–62. 10.1016/j.coi.2006.11.008 17137775

[22] Jensen LT. The aminoterminal propeptide of type III procollagen. Studies on physiology and pathophysiology. Dan Med Bull. 1997;44(1):70–8. 9062765

[23] Canty EG, Kadler KE. Procollagen trafficking, processing and fibrillogenesis. J Cell Sci. 2005;118(Pt 7):1341–53. 10.1242/jcs.01731 15788652

[24] Kirk JM, Bateman ED, Haslam PL, Laurent GJ, Turner-Warwick M. Serum type III procollagen peptide concentration in cryptogenic fibrosing alveolitis and its clinical relevance. Thorax. 1984;39(10):726–32. 6495240 10.1136/thx.39.10.726 PMC459910

[25] Low RB, Giancola MS, King TE Jr., Chapitis J, Vacek P, Davis GS. Serum and bronchoalveolar lavage of N-terminal type III procollagen peptides in idiopathic pulmonary fibrosis. Am Rev Respir Dis. 1992;146(3):701–6. 10.1164/ajrccm/146.3.701 1519851

[26] Entzian P, Huckstadt A, Kreipe H, Barth J. Determination of serum concentrations of type III procollagen peptide in mechanically ventilated patients. Pronounced augmented concentrations in the adult respiratory distress syndrome. Am Rev Respir Dis. 1990;142(5):1079–82. 10.1164/ajrccm/142.5.1079 2240830

[27] Meduri GU, Tolley EA, Chinn A, Stentz F, Postlethwaite A. Procollagen types I and III aminoterminal propeptide levels during acute respiratory distress syndrome and in response to methylprednisolone treatment. Am J Respir Crit Care Med. 1998;158(5 Pt 1):1432–41. 9817690 10.1164/ajrccm.158.5.9801107

[28] Deheinzelin D, Jatene FB, Saldiva PH, Brentani RR. Upregulation of collagen messenger RNA expression occurs immediately after lung damage. Chest. 1997;112(5):1184–8. 9367455 10.1378/chest.112.5.1184

[29] Pugin J, Verghese G, Widmer MC, Matthay MA. The alveolar space is the site of intense inflammatory and profibrotic reactions in the early phase of acute respiratory distress syndrome. Crit Care Med. 1999;27(2):304–12. 10075054 10.1097/00003246-199902000-00036

[30] Armstrong L, Thickett DR, Mansell JP, Ionescu M, Hoyle E, Billinghurst RC et al Changes in collagen turnover in early acute respiratory distress syndrome. Am J Respir Crit Care Med. 1999;160(6):1910–5. 10.1164/ajrccm.160.6.9811084 10588605

[31] Marshall RP, Bellingan G, Webb S, Puddicombe A, Goldsack N, McAnulty RJ et al Fibroproliferation occurs early in the acute respiratory distress syndrome and impacts on outcome. Am J Respir Crit Care Med. 2000;162(5):1783–8. 10.1164/ajrccm.162.5.2001061 11069813

[32] Clark JG, Milberg JA, Steinberg KP, Hudson LD. Type III procollagen peptide in the adult respiratory distress syndrome. Association of increased peptide levels in bronchoalveolar lavage fluid with increased risk for death. Ann Intern Med. 1995;122(1):17–23. 7985891 10.7326/0003-4819-122-1-199501010-00003

[33] Chesnutt AN, Matthay MA, Tibayan FA, Clark JG. Early detection of type III procollagen peptide in acute lung injury. Pathogenetic and prognostic significance. Am J Respir Crit Care Med. 1997;156(3 Pt 1):840–5. 9310002 10.1164/ajrccm.156.3.9701124

[34] Forel JM, Guervilly C, Hraiech S, Voillet F, Thomas G, Somma C et al Type III procollagen is a reliable marker of ARDS-associated lung fibroproliferation. Intensive Care Med. 2015;41(1):1–11. 10.1007/s00134-014-3524-0 25354475

[35] The ARDS Definition Task Force, Ranieri VM, Rubenfeld GD, Thompson BT, Ferguson ND, Caldwell E et al Acute respiratory distress syndrome: the Berlin Definition. JAMA. 2012;307(23):2526–33. 10.1001/jama.2012.5669 22797452

[36] Vincent JL, Moreno R, Takala J, Willatts S, De Mendonca A, Bruining H et al The SOFA (Sepsis-related Organ Failure Assessment) score to describe organ dysfunction/failure. On behalf of the Working Group on Sepsis-Related Problems of the European Society of Intensive Care Medicine. Intensive Care Med. 1996;22(7):707–10. 8844239 10.1007/BF01709751

[37] Blobe GC, Schiemann WP, Lodish HF. Role of transforming growth factor beta in human disease. N Engl J Med. 2000;342(18):1350–8. 10.1056/NEJM200005043421807 10793168

[38] Sporn MB. The early history of TGF-beta, and a brief glimpse of its future. Cytokine Growth Factor Rev. 2006;17(1–2):3–7. 10.1016/j.cytogfr.2005.09.012 16290110

[39] Heine UI, Munoz EF, Flanders KC, Roberts AB, Sporn MB. Colocalization of TGF-beta 1 and collagen I and III, fibronectin and glycosaminoglycans during lung branching morphogenesis. Development. 1990;109(1):29–36. 2209468 10.1242/dev.109.1.29

[40] Sporn MB, Roberts AB, Shull JH, Smith JM, Ward JM, Sodek J. Polypeptide transforming growth factors isolated from bovine sources and used for wound healing in vivo. Science. 1983;219(4590):1329–31. 6572416 10.1126/science.6572416

[41] Wahl SM. Transforming growth factor beta: the good, the bad, and the ugly. J Exp Med. 1994;180(5):1587–90. 7964446 10.1084/jem.180.5.1587 PMC2191721

[42] Buhling F, Tholert G, Kaiser D, Hoffmann B, Reinhold D, Ansorge S et al Increased release of transforming growth factor (TGF)-beta1, TGF-beta2, and chemoattractant mediators in pneumonia. J Interferon Cytokine Res. 1999;19(3):271–8. 10.1089/107999099314207 10213466

[43] Coker RK, Laurent GJ, Shahzeidi S, Lympany PA, du Bois RM, Jeffery PK, McAnulty RJ. Transforming growth factors-beta 1, -beta 2, and -beta 3 stimulate fibroblast procollagen production in vitro but are differentially expressed during bleomycin-induced lung fibrosis. Am J Pathol. 1997 3;150(3):981–91. 9060836 PMC1857875

