# Supplementary material for: Transforming Growth Factor-β1 in predicting early lung fibroproliferation in patients with acute respiratory distress syndrome
Source: PLoS One. 2018 Nov 5;13(11):e0206105. doi: 10.1371/journal.pone.0206105 (PMC6218031; doi:10.1371/journal.pone.0206105)

A. ROC Alveolar TGF β-1 on day 3 to fibroproliferation

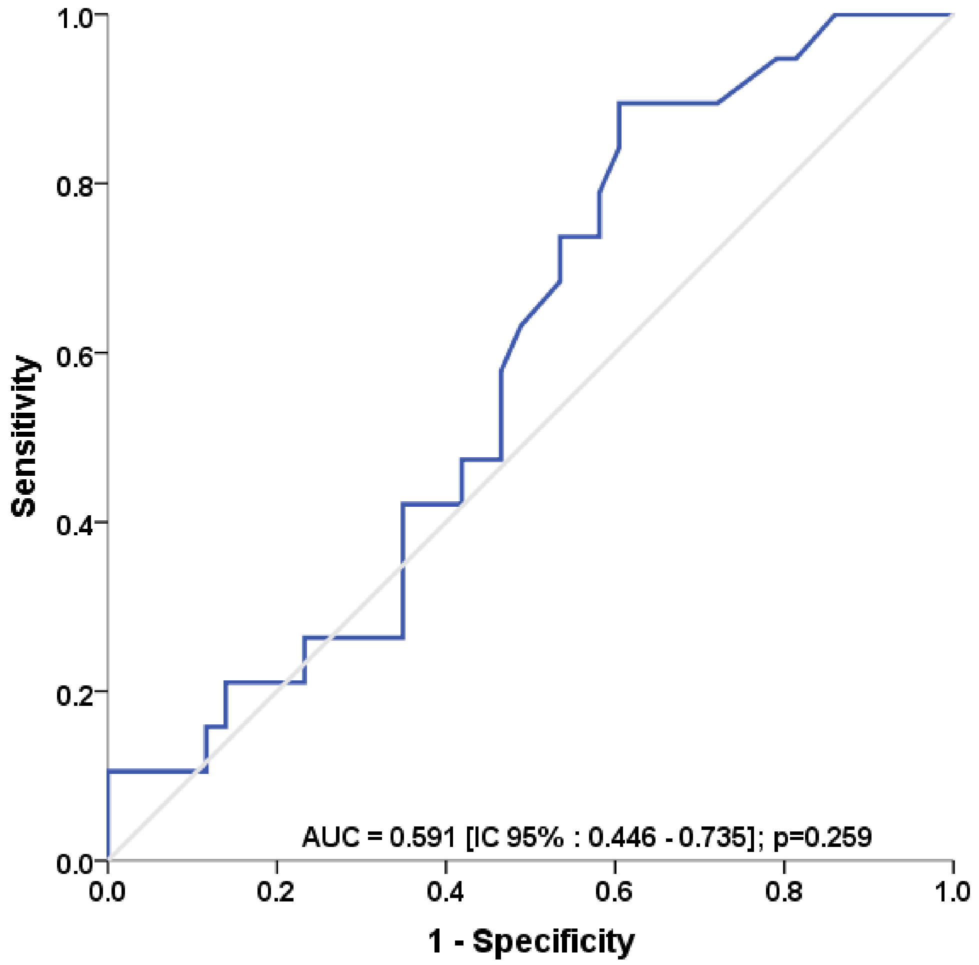

B. ROC Alveolar TGF β-1 on day 7 to fibroproliferation

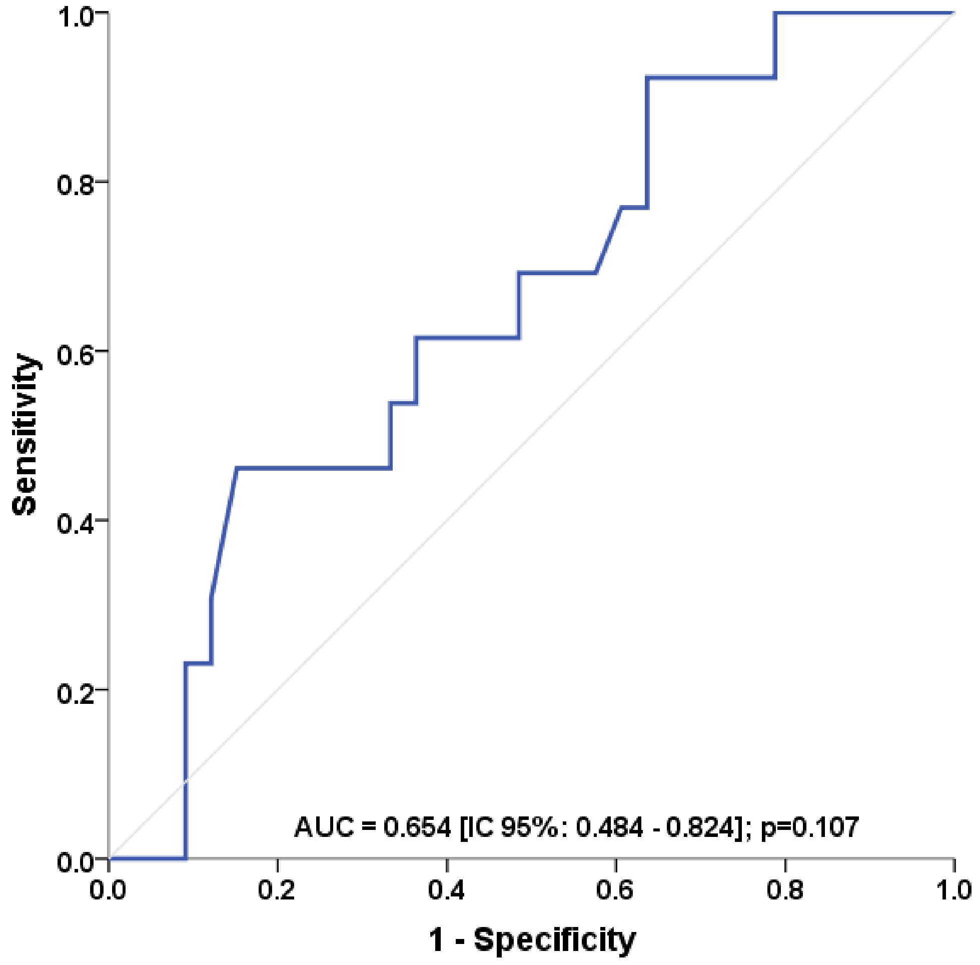

Supplement: S1 Fig — (PDF) [file pone.0206105.s001.pdf]
