# Supplementary figures and images for: Transforming Growth Factor-β1 in predicting early lung fibroproliferation in patients with acute respiratory distress syndrome
Source: PLoS One. 2018 Nov 5;13(11):e0206105. doi: 10.1371/journal.pone.0206105 (PMC6218031; doi:10.1371/journal.pone.0206105)

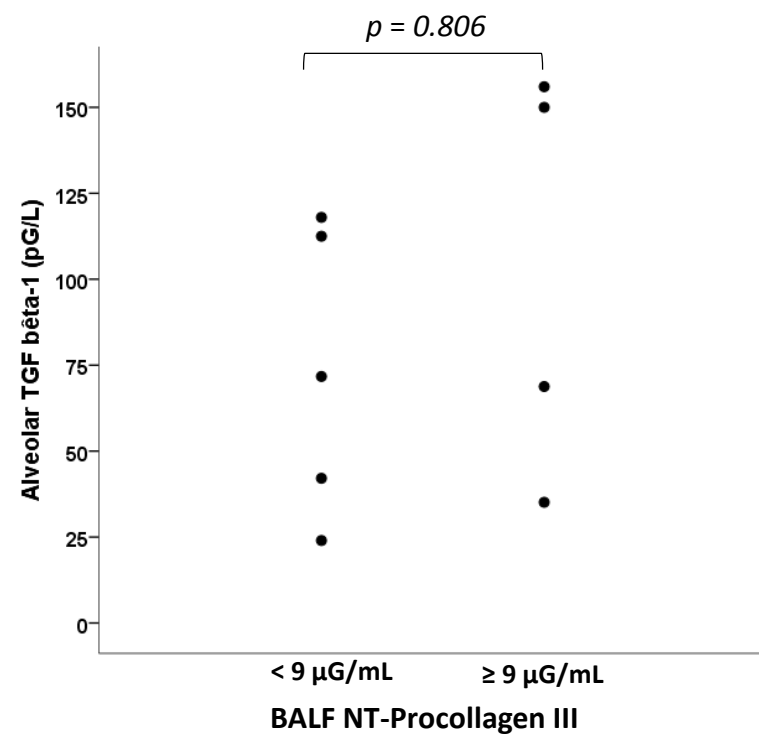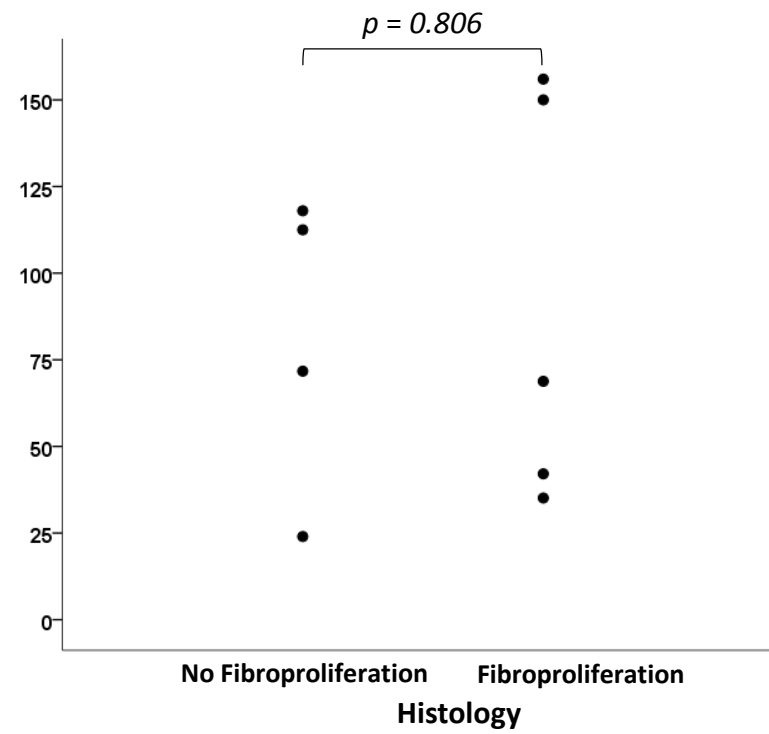

Supplement: S2 Fig — Bronchoalveolar lavage fluid levels of TGF-β1 in ARDS patients according to lung fibroproliferation evaluated by the level of alveolar NT-PCP-III (left panel) and histology (right panel). (PDF) [file pone.0206105.s002.pdf]

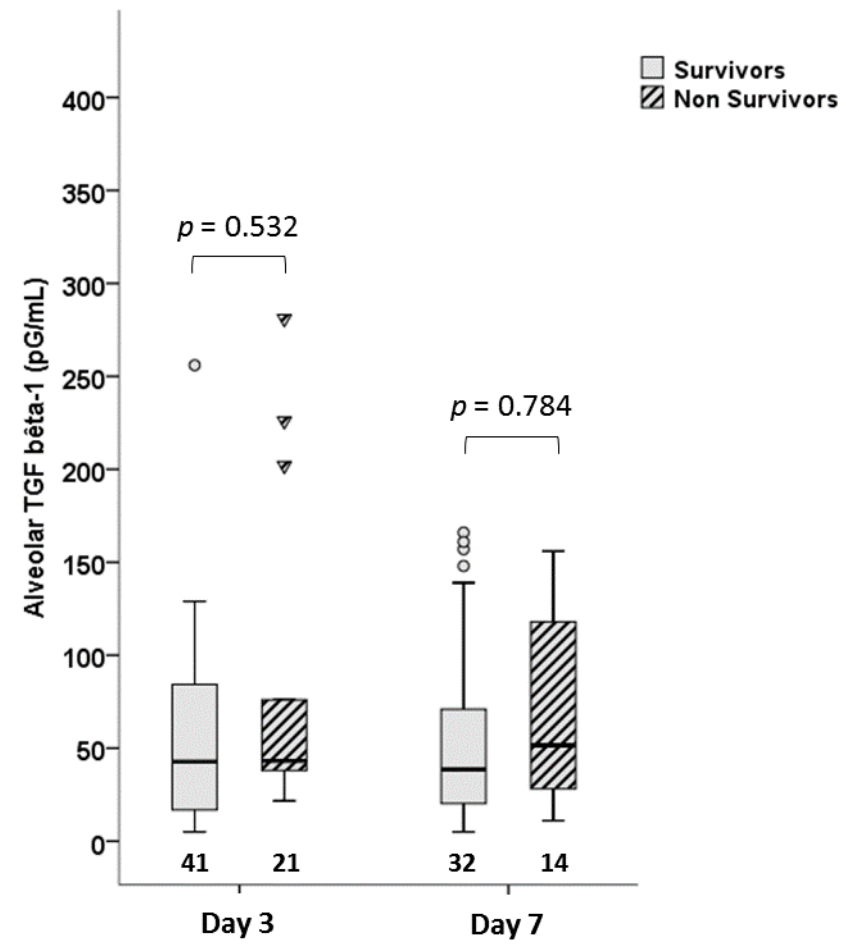

Supplement: S3 Fig — (PDF) [file pone.0206105.s003.pdf]
