# Supplementary material for: Transforming Growth Factor-β1 in predicting early lung fibroproliferation in patients with acute respiratory distress syndrome
Source: PLoS One. 2018 Nov 5;13(11):e0206105. doi: 10.1371/journal.pone.0206105 (PMC6218031; doi:10.1371/journal.pone.0206105)
Supplement: S1 Table — (DOCX) [file pone.0206105.s004.docx]

**ESM Table 1. Alveolar Free TGF β-1 according to the presence or not of lung fibroproliferation**

|  | **No fibroproliferation** | **Fibroproliferation** | ***p* value** |
| --- | --- | --- | --- |
| BALF Free TGF β-1 on day 3, pG/mL | 2 [2-2] | 2 [2-2] | 0.17 |
| BALF Free TGF β-1 on day 7, pG/mL | 2 [2-2] | 2 [2-2] | 0.10 |

Fibroproliferation was determined by a level of BALF NT-procollagen III level on day 3 or on day 7 upper than 9 µG/L. 62 patients were analyzed on day 3 (43 without fibroproliferation and 19 with fibroproliferation). 46 patients were analyzed on day 7 (33 without fibroproliferation and 13 with fibroproliferation). Values are presented as median [IQR]. BALF: broncho-alveolar lavage fluid.
